# Relationships among Fecal, Air, Oral, and Tracheal Microbial Communities in Pigs in a Respiratory Infection Disease Model

**DOI:** 10.3390/microorganisms9020252

**Published:** 2021-01-27

**Authors:** Robert Valeris-Chacin, Amanda Sponheim, Eduardo Fano, Richard Isaacson, Randall S. Singer, Joel Nerem, Fernando L. Leite, Maria Pieters

**Affiliations:** 1 Department of Veterinary and Biomedical Sciences, College of Veterinary Medicine, University of Minnesota, Saint Paul, MN 55108, USA; valer080@umn.edu (R.V.-C.); isaac015@umn.edu (R.I.); rsinger@umn.edu (R.S.S.); 2 Department of Veterinary Population Medicine, College of Veterinary Medicine, University of Minnesota, Saint Paul, MN 55108, USA; nessx317@umn.edu; 3 Boehringer Ingelheim Animal Health, Duluth, GA 30096, USA; eduardo.fano@boehringer-ingelheim.com; 4 Pipestone Applied Research, Pipestone, MN 56164, USA; joel.nerem@pipestone.com

**Keywords:** swine, *Mycoplasma hyopneumoniae*, fecal, tracheal, environment, microbiome

## Abstract

The association of the lower respiratory tract microbiome in pigs with that of other tissues and environment is still unclear. This study aimed to describe the microbiome of tracheal and oral fluids, air, and feces in the late stage of *Mycoplasma hyopneumoniae* infection in pigs, and assess the association between the tracheal microbiome and those from air, feces, and oral fluids. Tracheal fluids (n = 73), feces (n = 71), oropharyngeal fluids (n = 8), and air (n = 12) were collected in seeder pigs (inoculated with *M. hyopneumoniae*) and contact pigs (113 days post exposure to seeder pigs). After DNA extraction, the V4 region from 16S rRNA gene was sequenced and reads were processed using Divisive Amplicon Denoising Algorithm (DADA2). *Clostridium* and *Streptococcus* were among the top five genera identified in all sample types. *Mycoplasma hyopneumoniae* in tracheal fluids was associated with a reduction of diversity and increment of *M. hyorhinis*, *Glaesserella parasuis*, and *Pasteurella multocida* in tracheal fluids, as well as a reduction of *Ruminiclostridium, Barnesiella,* and *Lactobacillus* in feces. Air contributed in a greater proportion to bacteria in the trachea compared with feces and oral fluids. In conclusion, evidence suggests the existence of complex interactions between bacterial communities from distant and distinct niches.

## 1. Introduction

The collection of microorganisms and their genetic elements, persistently or transiently present in a certain ecological niche, is known as its microbiome. The microbiome can be decomposed in different dimensions depending on the type of microorganisms of interest, e.g., the viral microbiome (virome), the fungal microbiome (mycobiome), or the bacterial microbiome. The latter is the most commonly studied and is associated with a plethora of effects in animal hosts, such as breakdown of otherwise indigestible feedstuff (influencing feed efficiency [1]), competitive exclusion of pathogens [2], priming and regulation of the mucosal immune system [3,4], and disease [5].

The most studied microbiome by far is the gut microbiome. Each segment of the gut has a specific associated microbiome, which can be shaped by diet, host genetics, and environmental factors [6,7,8]. Zhao et al. [9] documented that aerobic and facultative anaerobic bacteria dominate in the small intestine of adult pigs, while anaerobic bacteria dominate in the large intestine. In pigs, the fecal microbiome is more related to the large intestine microbiome [9] and some of the genera commonly present in high abundance include *Bacteroides*, *Lactobacillus*, *Prevotella, Clostridium, Escherichia,* and *Megasphaera,* with evidence of bacterial succession during the pig’s life [10,11,12,13,14,15]. Changes in the fecal microbiome are associated with infections from major swine pathogens, such as *Salmonella*, *Lawsonia*, *Brachyspira*, and porcine epidemic diarrhea virus (PEDV) [16,17,18,19].

The microbiome of the oropharyngeal section of the digestive system in pigs is composed predominantly of *Streptococcus*, *Lactobacillus*, and *Actinobacillus* [20]. Shared between the digestive and respiratory systems, the oropharyngeal section is attractive for sampling due to their accessibility. In fact, oral fluids, saliva, and oral mucosal transudate [21] collected in ropes gnawed on by pigs in pens, are widely used for the detection of respiratory viruses in swine herds [22,23]. Although Murase, et al. [24] described the microbiome in pig saliva, the microbiome associated with oral fluids was not described.

The microbiome from certain sections of the respiratory tract in pigs was documented [15,25,26]. However, the extent of evidence is far less than that for the gut microbiome. More specifically, the lung microbiome in pigs affected by *Mycoplasma hyopneumoniae* was described to be dominated by bacteria members of the families *Mycoplasmataceae*, *Flavobacteriaceae*, and *Pasteurellaceae* if there are signs of pneumonia, or by members of *Mycoplasmataceae*, *Bradyrhizobiaceae*, and *Flavobacteriaceae* otherwise [25]. *Mycoplasma hyopneumoniae* is a major respiratory pathogenic bacterium in pigs, causing a chronic disease characterized by transient dry cough and increased susceptibility to other respiratory pathogens [27,28,29]. By contrast, the microbiome associated with the trachea in pigs has not yet been described, nor has its association with the extent of *M. hyopneumoniae* colonization. This is of interest, since deep tracheal catheter samples are nowadays commonly used for the detection of *M. hyopneumoniae* in live pigs, as the trachea represents an important site of colonization [30].

Recently, researchers strived to characterize the microbiome of the environment of swine facilities [31,32,33,34]. The air microbiome is of special interest as it may serve as an epidemiological vehicle for the transmission of microorganisms between different environments. A core of 19 bacterial families were shown to increase in the air microbiome of swine farms and in the noses of pigs and workers in comparison with the microbiome of the noses of people not exposed to live pigs [32].

Currently, there is little evidence of the association of the microbiome residing in the lower respiratory tract in pigs with that of other environments. The lower respiratory tract has long been considered a pristine and privileged section of the host, regarding bacterial colonization. However, recent data uncovered microbial communities that colonize the lower respiratory tract and appear to influence host responses to disease. Therefore, the objectives of this study were to describe the microbiome associated with tracheal and oral fluids, air, and feces during late stages of *M. hyopneumoniae* infection in pigs, to assess their potential association with infection status, and to infer to what extent the tracheal microbiome is related to microbial communities from air, feces, and oral fluids.

## 2. Materials and Methods

All pigs in this study were cared for following the guidelines of the Institutional Animal Care and Use Committee of Pipestone Applied Research (IACUC #3–18). Crossbred pigs were sourced from a commercial sow farm with high health status, known to be positive for influenza A virus, and negative for Porcine Reproductive and Respiratory Syndrome Virus (PRRSV) and *M. hyopneumoniae* infection for the last five years. Pigs were weaned at approximately three weeks of age, then transported and placed in a Midwestern United States commercial wean-to-finish facility designed for research [30]. Pigs were separated into groups of seeders (experimentally infected) and contacts (naturally infected via contact with seeders) via systematic assignment after blocking by sow parity range and ordering them by sex and weight. Seeders and contacts were randomly allocated to pens based on a 1:6 seeder-to-contact ratio, for a total of 28 pigs per pen. At eight weeks of age, seeders were intratracheally inoculated with *M. hyopneumoniae* and contacts were exposed to seeders following inoculation [30]. Samples included in this study were collected randomly from a subset of seeders (n = 47) and contacts (n = 26), or the environment at 113 days postexposure to *M. hyopneumoniae*. The majority of contact pigs were confirmed infected at least one month prior to sampling. Therefore, they were considered to be at a late infection stage [24]. Pigs were not exposed to antimicrobials known to be effective against *Mycoplasma* species during the study. Five out of the 73 selected pigs (one seeder and four contacts) were treated for clinical signs suggestive of *Streptococcus suis* infection using penicillin or ceftiofur, as indicated, at least two months prior to sample collection.

Deep tracheal catheters were used to collect tracheal fluids in pigs [30]. Fecal samples were obtained by digital insertion immediately following tracheal fluid sample collection. Oral fluid samples (n = 8) were collected as described by Prickett et al. [23], a procedure standardized to be performed at the pen level. Briefly, cotton ropes were hung using new gloves on the front gate of pens at pig shoulder height and pigs were allowed to chew on the ropes for 30 min. New gloves were donned for each rope collection and a new Ziploc bag was placed around the rope. The rope was squeezed from outside of the Ziploc bag while pulling away from the wall hook. The oral fluid sample was poured off from the Ziploc bag into a sterile snap cap Falcon tube, without touching the sample. Pens for sample collection were selected using a random number generator.

Six cyclonic filter collection devices (ACD-200 BOBCAT Air Sampler, Drexel, MO, USA) distributed throughout the entire air space where pens were located were used to collect air samples (n = 12). Air samples were collected for one hour during each of two samplings. Once the filtration run was complete, the filter was removed and rinsed using the manufacturer provided filter reagent into a sterile cup. All samples were refrigerated immediately and submitted to the Health Management Center (HMC; Boehringer Ingelheim Animal Health, Ames, IA, USA) for analysis.

DNA extraction for oral fluid and air samples (MagMAX^TM^ Pathogen DNA/RNA Kit, Life Technologies, Grand Island, NY, USA) [35,36,37] or tracheal fluid samples (MagMax-96 Viral RNA Isolation Kit, Life Technologies, Grand Island, NY, USA) [38] was performed using an extraction robot (KingFisher^TM^, Life Technologies, Grand Island, NY, USA). A species-specific real-time PCR was run for *M. hyopneumoniae* detection (VetMAX^TM^ qPCR Master Mix and VetMAX^TM^ *M. hyopneumoniae* Reagents Kit, Life Technologies, Grand Island, NY, USA) using a Roche LightCycler^®^ (480 Roche Life Science, Indianapolis, IN, USA). Both extraction and PCR methods were performed at the HMC. Real-time PCR samples with a Ct value < 38 were considered positive for *M. hyopneumoniae*. The *M. hyopneumoniae* infection status of each pig was based on the real-time PCR result in tracheal fluid samples, as those were individual samples that could be matched to a pig, contrary to environment and pen-based samples, such as air and oral fluids.

Fecal samples were submitted to the University of Minnesota Genomic Center (UMGC) for DNA extraction (DNAeasy PowerSoil kit, Qiagen, Germantown, MD, USA) and sequencing. The V4 region from 16S rRNA gene was amplified for 25 cycles in fecal samples and for 30 cycles in tracheal fluid, air, and oral fluid samples to account for the expected lower biomass in the latter. Samples were also dual indexed during library preparation. Sequencing of the amplicons was performed using MiSeq 2 × 300 bp platform.

All bioinformatics analyses were performed using R version 3.6.2 [39]. The quality profiles of the reads for each sample were visually inspected separately for forward and reverse reads. Primers and adapters were trimmed from all reads (Trim Galore version 0.6.4_dev for adapter removal, URL: http://www.bioinformatics.babraham.ac.uk/projects/trim_galore/). The last 15 bases of forward reads and the last 50 bases of reverse reads were also trimmed. Denoising of reads, merging of paired reads, and removal of chimera were performed using the Divisive Amplicon Denoising Algorithm (DADA2) package (version 1.14.0) [40]. DADA2-formatted training fasta file derived from the Silva Project’s version 132 release [41] was used to assign taxonomy to the amplicon sequence variants (ASV) obtained from the previous steps. Amplicon sequence variants from Eukarya, chloroplasts, and mitochondria were removed. Similarly, ASVs present in less than 1% of the samples were removed. This last step was performed separately by sample type. Samples with fewer reads than negative DNA extraction controls were removed as well.

The inverse Simpson alpha diversity index was computed and used in linear regression models to assess its association with sample type and *M. hyopneumoniae* Ct values, either as a continuous variable or as a binary variable (*M. hyopneumoniae* status). Detection of *M. hyopneumoniae* was not evaluated in the fecal samples due to the intrinsic restricted respiratory nature of this bacterium. Thus, *M. hyopneumoniae* Ct values from the tracheal fluid samples were used for analysis.

The following beta diversity dissimilarity indices were computed: Bray–Curtis, Jaccard, Unifrac, weighted Unifrac, and Aitchison. The beta diversity dissimilarity indices were used in principal coordinate analysis (PCoA) plots to explore the clustering of samples. The association of beta diversity dissimilarity indices with *M. hyopneumoniae* status, seeder/contact status, sample type, and the interaction of sample type and *M. hyopneumoniae* status was evaluated in tracheal fluids and fecal samples, using PERMANOVA stratified by pig [42] when appropriate.

With the aim of assessing the association between the ASVs corresponding to *M. hyopneumoniae* with the rest of the ASVs in tracheal fluids, the counts for all ASVs determined as *M. hyopneumoniae* through the search of the NCBI 16S ribosomal RNA sequences database (Bacteria and Archaea) using the nucleotide BLAST tool were aggregated. The centered log relative abundance (CLR) for all ASVs was calculated. In order to select the ASVs more strongly associated with *M. hyopneumoniae*, an elastic net model was built using glmnetUtils and glmnet packages in R [43,44], selecting the alpha and lambda parameters via leave-one-out cross-validation (LOOC).

A similar penalized approach was used to assess the association of the ASVs corresponding to *M. hyopneumoniae* in tracheal fluids with the ASVs in paired fecal samples, as well as the association of *M. hyopneumoniae* Ct values in tracheal fluids with the ASVs in the same samples and with the ASVs in paired fecal samples. Furthermore, ASVs differentially abundant according to *M. hyopneumoniae* status were detected in tracheal and fecal samples using ANCOM package in R [45].

A Bayesian finite mixture model (SourceTracker package) [46] was used to infer the proportions of the tracheal fluid microbiome potentially originating from the other microbial communities, i.e., fecal, oral fluids, and air microbiome, as putative sources. The Kolmogorov–Smirnov test was used to assess whether the empirical cumulative distribution functions (ecdf) of the proportions inferred for each microbial community differed according to *M. hyopneumoniae* status.

## 3. Results

**Figure 1:**
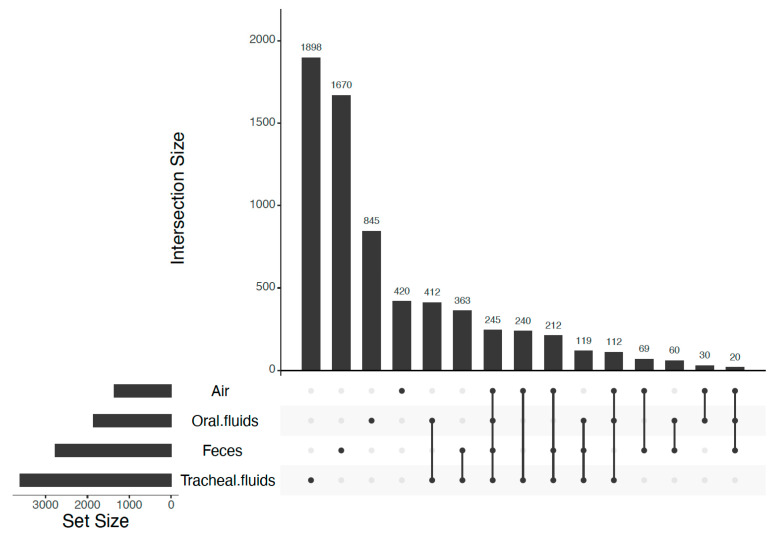
Amplicon sequence variants (ASVs) shared among sample types. Intersection size: number of ASVs comprising a specific nonoverlapping set. Dark circles indicate the sample types that are part of the set. Dark circles connected by a line indicate that two or more sample types are part of the set. The plot was made using UpSetR package in R [47] with the layout described by Lex and Gehlenborg [48].

The distribution of *M. hyopneumoniae*-positive samples categorized by sample type is shown in Table 1. Detection of *M. hyopneumoniae* was not evaluated in feces due to the respiratory nature of *M. hyopneumoniae* pathogenesis. The range and median of the sequencing depth (in reads) per sample type were 6079–23,346 and 15,486 for tracheal fluids, 4525–26,202 and 16,323 for air, 10,486–20,337 and 19,188 for oral fluids, and 6925–32,307 and 22,649 for feces, respectively. Rarefaction curves showed that the asymptote of the number of ASVs was reached in all sample types (Figure S1). The total numbers of detected ASVs were 3601 in tracheal fluids, 2758 in feces, 1843 in oral fluids, and 1348 in air. The range and median of ASVs detected per sample type were 115–368 and 229 ASVs for tracheal fluids, 65–371 and 263 ASVs for air, 104–362 and 276 ASVs for feces, and 217–397 and 371 ASVs for oral fluids, respectively. Overall, 26 percent of the ASVs detected in tracheal fluids were shared with feces, 25% with oral fluids, and 22% with air. The common presence of ASVs among sample types is represented in Figure 1. Furthermore, the set of ASVs exclusively shared between tracheal and oral fluids was the greatest nonoverlapping set (412 shared ASVs), followed by the set of ASVs exclusively shared between tracheal fluids and feces (363 shared ASVs). A set of shared ASVs, represented by those shared among all sample types, was composed of 245 ASVs. *Clostridium* and *Streptococcus* were consistently among the top five most abundant bacterial genera in all sample types (Table 2, Figures S2–S6).

**Figure 2:**
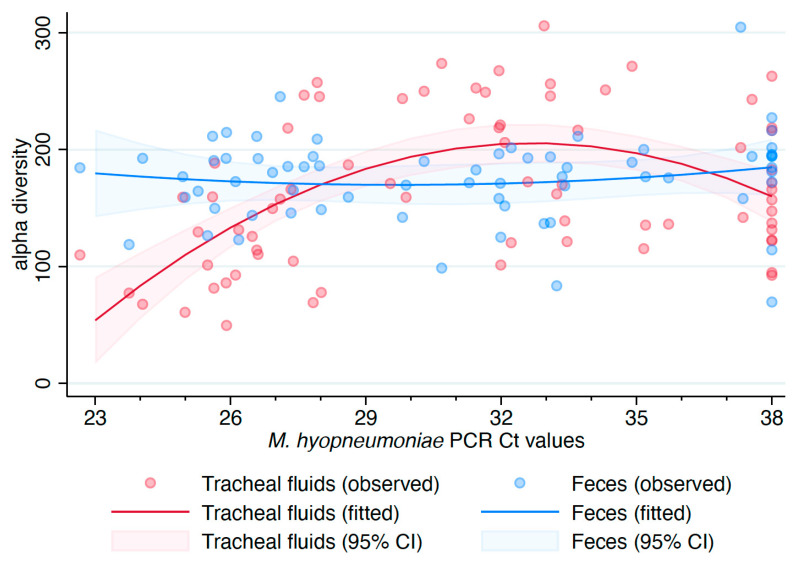
Association of alpha diversity (inverse Simpson index) with sample type and *Mycoplasma hyopneumoniae* real-time PCR Ct values in tracheal fluids. 95% CI: 95% confidence interval.

There was evidence of an overall effect of sample type on the alpha diversity (*p* = 0.0014), measured with the inverse Simpson index, when *M. hyopneumoniae* status was used as a covariate (Figure S7). No statistically significant overall effect for *M. hyopneumoniae* status was observed (*p* = 0.1672). Both overall effects were statistically significant when *M. hyopneumoniae* Ct values were used as covariate values instead (*p* < 0.0001 for each overall effect). Since *M. hyopneumoniae* DNA was detected in one of eight oral fluid samples and undetected in air samples, the linear model assessing the association among alpha diversity, sample types, and *M. hyopneumoniae* Ct values was reduced to include tracheal fluids and fecal samples. No association between alpha diversity of fecal samples and *M. hyopneumoniae* Ct values in tracheal fluids was observed (*p* = 0.6538). However, a quadratic association between alpha diversity of tracheal fluids and *M. hyopneumoniae* Ct values was detected (*p* < 0.0001), with a faster reduction in alpha diversity as the Ct values decreased (Figure 2).

**Figure 3:**
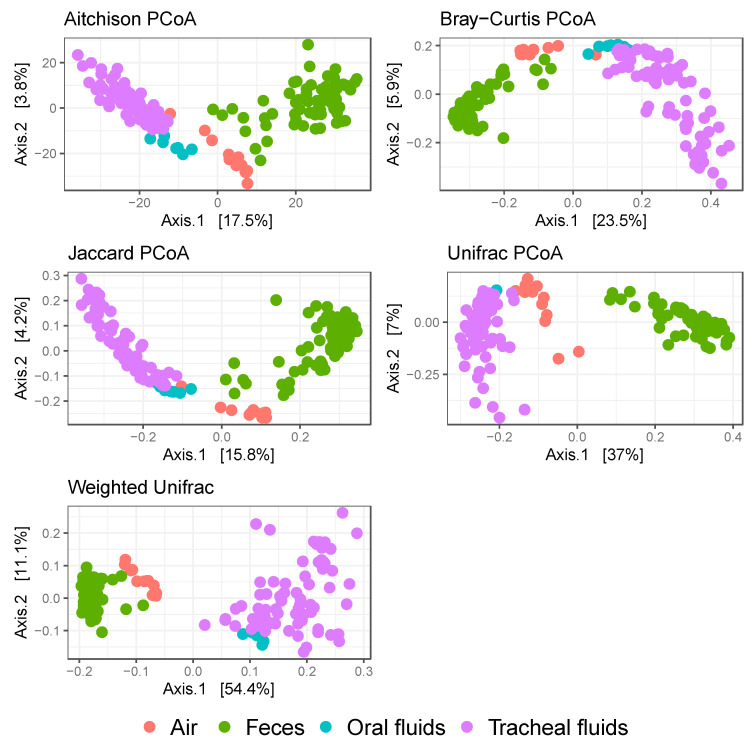
Microbial communities clustering analysis. Each dot represents an individual sample. Five different dissimilarity indices were performed and are shown in the following order (left to right, top to bottom): Aitchison, Bray–Curtis, Jaccard, Unifrac, and weighted Unifrac. Samples are color-coded by type. Orange: air; green: feces; teal: oral fluids; purple: tracheal fluids.

All sample types were analyzed using several dissimilarity measures in PCoA plots for the assessment of clustering, namely Aitchison, Bray–Curtis, Jaccard, Unifrac, and weighted Unifrac. Three clusters were consistently distinguished in the PCoA plots, one cluster composed of fecal samples, another composed of air samples, and a third cluster composed of tracheal and oral fluid samples (Figure 3). A significant association between sample type and beta diversity was observed irrespective of the dissimilarity index used (Table 3). *Mycoplasma hyopneumoniae* status was significantly associated with beta diversity in tracheal fluids, except when the Unifrac index was used. However, *M. hyopneumoniae* was not significantly associated with beta diversity in fecal samples regardless of the index used. Similarly, *M. hyopneumoniae* modified the association between sample type and beta diversity when either Bray–Curtis or the weighted Unifrac index were used (Table 3). Seeder/contact status was not significantly associated with beta diversity in tracheal fluids or fecal samples, except when the weighted Unifrac index was used in tracheal fluids.

**Figure 4:**
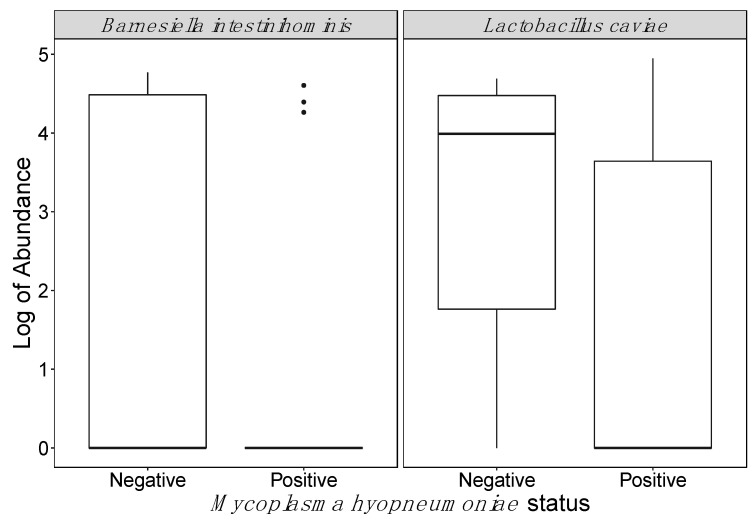
Amplicon sequence variants differentially abundant in fecal samples according to *Mycoplasma hyopneumoniae* status of paired tracheal fluid samples. Real-time PCR in tracheal samples was used to establish *M. hyopneumoniae* status.

A total of 205 ASVs in tracheal fluids were associated with *M. hyopneumoniae* ASVs in tracheal fluid samples (Table S1). One ASV belonging to the genus *Ruminiclostridium* in feces displayed a negative association with *M. hyopneumoniae* ASVs in tracheal fluids (Figure S8). Two ASVs in tracheal fluids, from genera *Clostridium* and *Parabacteroides*, were positively associated with *M. hyopneumoniae* PCR Ct values, whereas two ASVs from the genus *Pasteurella* and the ASVs corresponding to *M. hyopneumoniae* in tracheal fluids were negatively associated with Ct values (Figures S9–S12). No ASVs in feces were associated with *M. hyopneumoniae* PCR Ct values in tracheal fluids. Furthermore, no ASVs in tracheal fluids were significantly associated with *M. hyopneumoniae*-positive status, except for those belonging to *M. hyopneumoniae*. In contrast, two ASVs from the genera *Barnesiella* and *Lactobacillus* in feces were shown to have a significant negative association with *M. hyopneumoniae*-positive status (FDR = 0.05; Figure 4). *Mycoplasma hyopneumoniae* ASVs were not detected in feces.

**Figure 5:**
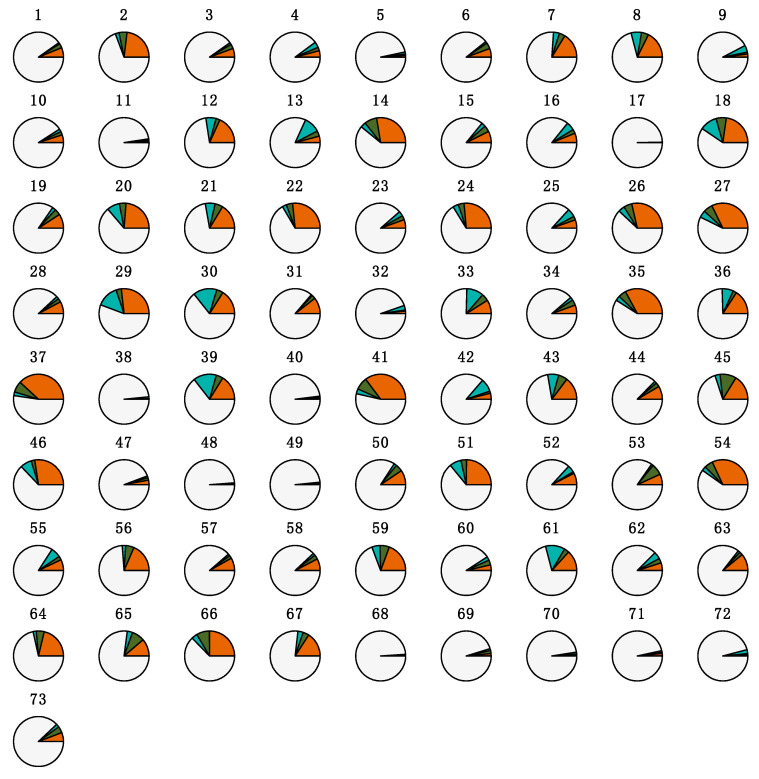
Proportions of the tracheal fluid microbial community potentially originating from fecal, air, or oral fluid sources. Each pie represents the tracheal fluid of one individual pig (n = 73). Sources are color-coded. Orange: air; green: feces; teal: oral fluids; light gray: unknown.

The heterogeneity in the proportions of the tracheal fluid microbial community potentially originating from fecal, air, or oral fluids sources is depicted in Figure 5. The characteristics of the distribution of the estimated proportions of ASVs by source are shown in Table 4. There was no statistical evidence that the distribution of the proportions of ASVs potentially originating from each microbial community differed according to *M. hyopneumoniae* status (*p =* 0.1815, 0.9903, and 0.4273 for air, fecal, and oral fluid sources, respectively; Figures S13–S15).

## 4. Discussion

This study described the microbiome identified in tracheal and oral fluids, air, and feces of pigs reared under commercial conditions during late-stage infection with *M. hyopneumoniae*. Results of this study showed that both alpha and beta diversity significantly differed among sample types. The presence of *M. hyopneumoniae* in tracheal samples was associated with several bacterial species in those samples, as well as in fecal samples. Moreover, this research assessed whether the tracheal microbiome could originate from microbial communities from air, feces, and oral fluids in pigs. Our results suggest that a relatively small but higher proportion of the tracheal microbiome community may have originated from the air, compared to the other two studied potential sources, namely, feces and oral fluids.

Infections caused by *M. hyopneumoniae* are highly prevalent worldwide and constitute the most important bacterial disease affecting the respiratory tract in swine [29]. Pigs affected with *M. hyopneumoniae* generally do not succumb to the disease. However, they remain infectious for extremely long periods, reaching up to seven months [28], which identifies this infection as chronic in nature, leading to endemic situations. Infected pigs usually exhibit growth retardation and increased susceptibility to other respiratory pathogens of bacterial and viral origin [29]. Thus, *M. hyopneumoniae* is a key player in the development of the porcine respiratory disease complex. Therefore, the *M. hyopneumoniae* infection model recreated in this study is considered ideal to assess the effect of bacterial infections in the respiratory microbial communities.

*Clostridium* and *Streptococcus* were consistently among the top five most abundant bacterial genera in all sample types. *Streptococcus* is commonly documented in several pig niches. *Streptococcus* is part of the core tonsillar microbiome in newborn piglets [26], and is also part of the core oropharyngeal microbiome in piglets, together with *Lactobacillus* and *Actinobacillus* [20]. *Streptococcus* is one of the most abundant genera in pig saliva, along with *Actinobacillus, Moraxella*, and *Rothia* [24], and was reported as the major bacterial genus in aerosols from pig confinement buildings [33]. Additionally, *Clostridium* and *Escherichia* were documented to be among the most abundant genera in feces during the preweaning phase, while *Megasphaera* and *Lactobacillus* seem to be more abundant during the postweaning phase in pigs [12,13,14,15]. *Clostridium* was also shown to have a high relative abundance in the noses of pigs before weaning, being replaced by *Lactobacillus* thereafter [15].

The alpha diversity of a bacteriological niche is comprised of the number of bacterial species and the evenness of the distribution of those species [49]. The alpha diversity of the tracheal microbiome decreased with greater concentrations of *M. hyopneumoniae*, which is in agreement with other studies, where pathogens, such as PEDV in pigs and *Enterococcus faecalis* in humans, induce the reduction of the evenness and richness in their ecological niche [50,51]. The dominance of *M. hyopneumoniae* in the tracheas of affected pigs could have led to an imbalance of the proportions of the bacterial members of the tracheal microbiome, resulting in a lower evenness and alpha diversity in comparison with the trachea of pigs not infected with this bacterium. *Mycoplasma hyopneumoniae*-induced shift in the tracheal microbiome alpha diversity may not only reflect its fitness at colonizing the tracheal epithelium, but also its ability to modulate the microbiome to its advantage.

Differences in the microbiome composition among samples collected in this study were reflected in the association of beta diversity and sample type, which was robust to the dissimilarity index used. Similarly, beta diversity was shown to reflect age effects in the nasal and fecal microbiomes of pre- and postweaning pigs [15], and litter effects in the tonsillar microbiomes of newborn piglets [26].

Beta diversity is intrinsically related to differentially abundant bacteria. Using different approaches to parameterize the presence of *M. hyopneumoniae* in tracheal samples, *M. hyorhinis*, *Niastella hibisci* [52], *Glaesserella parasuis*, *Terrimonas rubra* [53], and *Pasteurella multocida* were observed to increase in tracheal samples alongside *M. hyopneumoniae*. It is remarkable that *M. hyopneumoniae* was associated with three major swine pathogens. *Mycoplasma hyorhinis* may cause arthritis and/or polyserositis [54], *Glaesserella* (*Haemophilus*) *parasuis* is the etiologic agent of Glässer’s disease characterized by polyserositis [55], and *P. multocida* is associated with atrophic rhinitis and the porcine respiratory disease complex [56]. However, all three bacteria can be present in the respiratory tracts of healthy pigs, which highlights the complexity of the pathogen–host-microbiome interaction. Furthermore, these findings support the current perception of *M. hyopneumoniae* as a pathogen capable of inducing dysbiosis in the respiratory tract, potentiating opportunistic pathogens in situ.

Negative associations were detected between *M. hyopneumoniae* in tracheal samples and bacteria from the genera *Ruminiclostridium, Barnesiella,* and *Lactobacillus* in fecal samples from the same pigs. *Ruminiclostridium* is member of the family *Ruminococcaceae*. Interestingly, the relative abundance of *Ruminococcus*, another genus of the family *Ruminococcaceae*, in feces was previously documented to be negatively associated with severity of lung lesions caused by *M. hyopneumoniae* [57]. Since *Barnesiella* and *Lactobacillus* are associated with a healthy gut microbiome [58,59], the findings of this study suggest that *M. hyopneumoniae*-induced dysbiosis in the respiratory tract microbiome may be associated through yet unknown mechanisms to changes in gut bacterial colonization patterns.

The analysis using a Bayesian finite mixture model allowed the estimation of the proportions of the tracheal microbial community originating from fecal, air, or oral fluid sources [46], providing deeper insights than analyses based on the presence/absence of ASVs and PCoA of the Jaccard dissimilarity index. Despite great heterogeneity, the air microbial community was more closely related to that of the trachea, hence, the estimated proportion for air as a source was greater than that inferred for feces and oral fluids. However, the distribution of the proportions was different from that observed in previous research [31], in which the inferred contribution of air as a source of the nasal microbiome in pigs and workers was higher than 40% throughout the year. Those differences could stem from the fact that Kraemer, Aebi, Oppliger, and Hilty [31] sampled the upper respiratory tract of pigs instead of the lower respiratory tract, as in this study.

This was a cross-sectional study, which was not suited to address the temporality of the associations under scrutiny. Since the type of samples collected was part of the design, the authors consider the effect of sample type on the microbiome not to be prone to confounding, i.e., bias due to common causes of the type of samples collected and the microbiome composition. Conversely, *M. hyopneumoniae* status at day 113 post contact was not randomized, but followed experimental infection in seeders and the inherent transmission of the pathogen in contacts, which could potentially affect the associations detected in this study between *M. hyopneumoniae* and microbiome composition. However, it is important to highlight that common confounders, such as age, breed, biosecurity practices, and diet, were similar for all pigs in the study. Therefore, the residual confounding was considered to be small. It is important to note that the differential number of 16S PCR cycles used to account for low biomass in air, oral, and tracheal fluid samples could have affected the number of ASVs detected in those specimens. This is especially relevant for comparisons between low (tracheal fluids) and high (feces) biomass specimens. Caution is warranted in the interpretation of the results from the Bayesian finite mixture model approach, since the directionality of the associations, referring to which samples are potential sources and which are the sink, are assumed to be known. In the case of this study, the authors assessed one possible scenario, in which the air, oral fluids, and feces were the potential sources and the tracheal microbiome was the sink. However, the true directionality is unknown. Moreover, the potential contribution of the microbiome from other sites of the respiratory tract, such as nasal microbiome, was not explored in this study. Future research assessing the contribution of nasopharyngeal microbiome to the bacterial communities of the lower respiratory tract is warranted.

An important aspect to mention is the fact that *M. hyopneumoniae* was mainly detected in the tracheal fluid samples, while remaining undetected in the majority of the oral fluid samples. This situation is commonly encountered in infection with this bacterium, even under experimental conditions [60], and may be related to the target tissue of the pathogen, which attaches to the cilia of the respiratory tract and may be detected in the oral cavity only after being coughed up by the host. For this reason, establishing the *M. hyopneumoniae* status of pigs was performed based on detection of the bacterium in tracheal fluid samples.

In recent years, a greater appreciation of the complex pathogen–host-microbiome interactions resulted from studies assessing the effects of the commensal bacterial microbiome not only in situ, but also outside its immediate ecological niche. For instance, protective effects of diverse fecal microbiome were shown against systemic viral diseases in pigs, specifically against PRRSV and PCV2 [61,62], and also against bacterial diseases, such as *M. hyopneumoniae* [63]. In this context, the findings of this study further our knowledge about the interplay between respiratory pathogens, *M. hyopneumoniae* in this case, and the local microbiome. More importantly, this study provides additional evidence of long-reaching effects of *M. hyopneumoniae* infection and the fecal microbiome. Future research should dissect these associations to better understand data directionality and temporality. In an era of increasing antibiotic stewardship, the prospect of manipulating the microbiome to prevent and/or mitigate infectious diseases fuels the momentum of the current microbiome research.

## 5. Conclusions

All studied bacterial niches, tracheal and oral fluids, air, and feces, showed intrinsic differences in their bacterial communities. However, *Clostridium* and *Streptococcus* were consistently among the top five most abundant bacterial genera across all bacterial niches. *Mycoplasma hyopneumoniae* infection status was associated with a shift in the tracheal microbiome, reducing its diversity and increasing the relative abundance of the major swine pathogens *M. hyorhinis*, *G. parasuis*, and *P. multocida.* Furthermore, *M. hyopneumoniae* infection was associated with a decrease in the relative abundance of *Ruminiclostridium, Barnesiella,* and *Lactobacillus* in feces. Finally, the air microbial community may have a greater contribution to the tracheal microbiome in comparison with feces and oral fluids.

## Figures and Tables

**Table 1:** Distribution of *Mycoplasma hyopneumoniae* detection in study fluids and samples.

Sample Type	*M. hyopneumoniae* Detection		Total Number of Samples
	Positive	Negative	
Tracheal fluids	59	14	73
Oral fluids	1	7	8
Air	0	12	12

**Table 2:** Five most abundant bacterial genera identified in microbial communities from various fluids and samples.

Rank/Sample Type	Tracheal Fluids		Feces	Oral Fluids	Air
	Positive *	Negative *			
1	*Mycoplasma* (14.1)	*Actinobacillus* (10.1)	*Clostridium* (24.3)	*Clostridium* (8.6)	*Clostridium* (32)
2	*Actinobacillus* (7.3)	*Streptococcus* (10.1)	*Akkermansia* (12.1)	*Terrisporobacter* (5.7)	*Terrisporobacter* (9.8)
3	*Streptococcus* (7.1)	*Clostridium* (7.5)	*Streptococcus* (12.1)	*Phascolarctobacterium* (5.5)	*Turicibacter* (9.1)
4	*Clostridium* (6.2)	*Prevotellaceae* NK3B31 group (4.2)	*Lactobacillus* (7.4)	*Leptotrichia* (5.4)	*Lactobacillus* (7.2)
5	*Prevotellaceae* NK3B31 group (3)	*Lactobacillus* (3.7)	*Turicibacter* (6.7)	*Streptococcus* (5)	*Streptococcus* (6.4)

* Positive or negative to *Mycoplasma hyopneumoniae* by real-time PCR. Median relative abundance (%) shown in parentheses.

**Table 3:** Association of beta diversity dissimilarity indices with sample type (tracheal fluids and feces) and the interaction of sample type and *Mycoplasma hyopneumoniae* status.

Effect/Dissimilarity Index	Aitchison	Bray–Curtis	Jaccard	Unifrac	Weighted Unifrac
Sample type					
R^2^	0.18	0.24	0.16	0.39	0.55
*p*	0.001	0.001	0.001	0.001	0.001
Interaction term					
R^2^	0.02	0.02	0.02	0.01	0.03
*p*	0.092	0.028	0.055	0.211	0.01

Interaction term: interaction between sample type and *M. hyopneumoniae* status. R^2^: coefficient of determination. Probability values (*p*) obtained via permutation.

**Table 4:** Characteristics of the distribution of the proportions of tracheal fluid microbial communities potentially originating from fecal, air, or oral fluid sources.

Source	Minimum	Median	Mean	Maximum
Air				
Positive	0.003	0.08	0.11	0.35
Negative	0.001	0.14	0.17	0.38
Overall	0.001	0.09	0.12	0.38
Feces				
Positive	0.001	0.03	0.03	0.1
Negative	0.001	0.04	0.04	0.08
Overall	0.001	0.03	0.04	0.1
Oral Fluids				
Positive	0.004	0.03	0.04	0.15
Negative	0.001	0.03	0.04	0.08
Overall	0.001	0.03	0.04	0.15

Samples segregated by *Mycoplasma hyopneumoniae* status as assessed in tracheal fluids.

## Data Availability

The data presented in this study are available on request from the corresponding authors. The data are not publicly available due to being proprietary to Boehringer Ingelheim Animal Health (BIAH).

## Supplementary Materials

The following are available online at https://www.mdpi.com/2076-2607/9/2/252/s1: Figures S1–S15, Table S1, File 1.txt: Top five results of the BLAST search for the ASVs associated with *M. hyopneumoniae* ASVs in tracheal fluid samples; File 2.txt: Top five results of the BLAST search for the ASVs in tracheal fluids associated with *M. hyopneumoniae* PCR Ct values; File 3.txt: Top five results of the BLAST search for the ASVs in feces associated with *M. hyopneumoniae*-positive status.
