# Supplementary material for: Relationships among Fecal, Air, Oral, and Tracheal Microbial Communities in Pigs in a Respiratory Infection Disease Model
Source: Microorganisms. 2021 Jan 27;9(2):252. doi: 10.3390/microorganisms9020252 (PMC7912642; doi:10.3390/microorganisms9020252)
Supplement: Supplementary file 1 [file microorganisms-09-00252-s001.zip › supplementary materials.docx]

**SUPPLEMENTARY MATERIALS**

**Figure S1.** Rarefaction curves by sample type before the removal of ASVs from Eukarya, chloroplasts, and mitochondria or of ASVs less than 1% prevalent. Rarefaction curves were created with ampvis2 package version 2.6.7 in R.

**Figure S2.** Top twenty most abundant bacterial genera in air.

**Figure S3**. Top twenty most abundant bacterial genera in tracheal fluids from pigs negative to *Mycoplasma hyopneumoniae*.

**Figure S4**. Top twenty most abundant bacterial genera in tracheal fluids from pigs positive to *Mycoplasma hyopneumoniae*.

**Figure S5.** Top twenty most abundant bacterial genera in feces.

**Figure S6.** Top twenty most abundant bacterial genera in oral fluids.

**Figure S7.** Adjusted means for alpha diversity (inverse Simpson index) by sample type adjusted by *Mycoplasma hyopneumoniae* status. Red vertical lines represent 95% confidence intervals.

**Figure S8.** Association of relative abundance, as centered log relative abundance (CLR), of an amplicon sequence variant belonging to the genus *Ruminoclostridium* in feces with the relative abundance of *Mycoplasma hyopneumoniae* in tracheal fluids. Scatter plot represents raw data. Fitted lines from a simple linear regression (in blue) and from a LOESS smoother (in red) are shown for reference.

**Figure S9.** Association of *Mycoplasma hyopneumoniae* PCR Ct values in tracheal fluids with the relative abundance, as centered log relative abundance (CLR), of an amplicon sequence variant belonging to the genus *Clostridium* in tracheal fluids. Scatter plot represents raw data. Fitted lines from a simple linear regression (in blue) and from a LOESS smoother (in red) are shown for reference.

**Figure S10.** Association of *Mycoplasma hyopneumoniae* PCR Ct values in tracheal fluids with the relative abundance, as centered log relative abundance (CLR), of an amplicon sequence variant belonging to the genus *Parabacteroides* in tracheal fluids. Scatter plot represents raw data. Fitted lines from a simple linear regression (in blue) and from a LOESS smoother (in red) are shown for reference.

**Figure S11.** Association of *Mycoplasma hyopneumoniae* PCR Ct values in tracheal fluids with the relative abundance, as centered log relative abundance (CLR), of two amplicon sequence variants belonging to the genus *Pasteurella* in tracheal fluids. Scatter plot represents raw data. Fitted lines from a simple linear regression (in blue) and from a LOESS smoother (in red) are shown for reference.

**Figure S12.** Association of *Mycoplasma hyopneumoniae* PCR Ct values in tracheal fluids with the relative abundance, as centered log relative abundance (CLR), of *M. hyopneumoniae* in tracheal fluids. Scatter plot represents raw data. Fitted lines from a simple linear regression (in blue) and from a LOESS smoother (in red) are shown for reference.

**Figure S13.** Empirical cumulative distribution function of the proportions of tracheal fluids microbiome attributable to air microbiome by *Mycoplasma hyopneumoniae* status.

**Figure S14.** Empirical cumulative distribution function of the proportions of tracheal fluids microbiome attributable to fecal microbiome by *Mycoplasma hyopneumoniae* status.

**Figure S15.** Empirical cumulative distribution function of the proportions of tracheal fluids microbiome attributable to oral fluids microbiome by *Mycoplasma hyopneumoniae* status.

**Table S1.** Bacterial species corresponding to the amplicon sequence variants (ASVs) associated with the relative abundance of *Mycoplasma hyopneumoniae* in tracheal fluids.

| BACTERIAL SPECIES | ASVs with positive association | ASVs with negative association | Total number of ASVs |
| --- | --- | --- | --- |
| *Acetatifactor muris* | 0 | 1 | 1 |
| *Acetitomaculum ruminis* | 0 | 1 | 1 |
| *Acinetobacter boissieri* | 0 | 1 | 1 |
| *Actinobacillus indolicus* | 0 | 8 | 8 |
| *Actinobacillus minor* | 0 | 4 | 4 |
| *Actinobacillus porcinus* | 0 | 10 | 10 |
| *Aerococcus suis* | 0 | 1 | 1 |
| *Aerococcus viridans* | 0 | 1 | 1 |
| *Akkermansia glycaniphila* | 0 | 1 | 1 |
| *Alloprevotella rava* | 0 | 5 | 5 |
| *Alysiella crassa* | 0 | 2 | 2 |
| *Anaerofilum pentosovorans* | 0 | 1 | 1 |
| *Anaerovibrio lipolyticus* | 0 | 2 | 2 |
| *Atopostipes suicloacalis* | 0 | 1 | 1 |
| *Bacteroides caecigallinarum* | 0 | 1 | 1 |
| *Bacteroides massiliensis* | 0 | 1 | 1 |
| *Blautia wexlerae* | 0 | 1 | 1 |
| *Butyricicoccus pullicaecorum* | 0 | 1 | 1 |
| *Caloramator fervidus* | 0 | 1 | 1 |
| *Campylobacter mucosalis* | 0 | 1 | 1 |
| *Carboxylicivirga mesophila* | 0 | 1 | 1 |
| *Catenibacterium mitsuokai* | 0 | 1 | 1 |
| *Chryseobacterium taklimakanense* | 0 | 3 | 3 |
| *Clostridium citroniae* | 0 | 1 | 1 |
| *Collinsella aerofaciens* | 0 | 1 | 1 |
| *Eggerthella lenta* | 0 | 1 | 1 |
| *Enorma phocaeensis* | 0 | 2 | 2 |
| *Enterorhabdus muris* | 0 | 1 | 1 |
| *Erysipelothrix inopinata* | 0 | 2 | 2 |
| *Eubacterium coprostanoligenes* | 0 | 2 | 2 |
| *Eubacterium eligens* | 0 | 1 | 1 |
| *Faecalibacterium prausnitzii* | 0 | 1 | 1 |
| *Flintibacter butyricus* | 0 | 2 | 2 |
| *Fournierella massiliensis* | 0 | 2 | 2 |
| *Fusobacterium gastrosuis* | 0 | 2 | 2 |
| *Gemella taiwanensis* | 0 | 1 | 1 |
| *Gemmiger formicilis* | 0 | 2 | 2 |
| *Glaesserella parasuis* | 5 | 1 | 6 |
| *Helicobacter rodentium* | 0 | 2 | 2 |
| *Holdemanella biformis* | 0 | 1 | 1 |
| *Holdemania filiformis* | 0 | 1 | 1 |
| *Hydrotalea flava* | 0 | 4 | 4 |
| *Ihubacter massiliensis* | 0 | 4 | 4 |
| *Intestinimonas butyriciproducens* | 0 | 1 | 1 |
| *Kingella denitrificans* | 0 | 1 | 1 |
| *Kroppenstedtia eburnea* | 0 | 1 | 1 |
| *Lachnoclostridium pacaense* | 0 | 4 | 4 |
| *Lachnospira pectinoschiza* | 0 | 1 | 1 |
| *Lactobacillus caviae* | 0 | 1 | 1 |
| *Lactobacillus leichmannii* | 0 | 2 | 2 |
| *Lactobacillus salivarius* | 0 | 1 | 1 |
| *Leptotrichia goodfellowii* | 0 | 3 | 3 |
| *Leptotrichia wadei* | 0 | 1 | 1 |
| *Massiliprevotella massiliensis* | 0 | 1 | 1 |
| *Megasphaera elsdenii* | 0 | 1 | 1 |
| *Monoglobus pectinilyticus* | 0 | 1 | 1 |
| *Moraxella porci* | 0 | 2 | 2 |
| *Muribaculum intestinale* | 0 | 5 | 5 |
| *Mycoplasma hyorhinis* | 2 | 0 | 2 |
| *Neisseria dentiae* | 0 | 1 | 1 |
| *Niastella hibisci* | 4 | 0 | 4 |
| *Oligosphaera ethanolica* | 0 | 2 | 2 |
| *Olsenella scatoligenes* | 0 | 1 | 1 |
| *Oscillibacter ruminantium* | 0 | 1 | 1 |
| *Paludibacter propionicigenes* | 0 | 1 | 1 |
| *Parabacteroides distasonis* | 0 | 1 | 1 |
| *Parapedobacter soli* | 0 | 1 | 1 |
| *Paraprevotella clara* | 0 | 2 | 2 |
| *Pasteurella aerogenes* | 0 | 1 | 1 |
| *Pasteurella multocida* | 17 | 0 | 17 |
| *Pleomorphochaeta caudata* | 0 | 1 | 1 |
| *Porphyromonas pasteri* | 0 | 1 | 1 |
| *Porphyromonas pogonae* | 0 | 1 | 1 |
| *Prevotella albensis* | 0 | 1 | 1 |
| *Prevotella buccalis* | 0 | 1 | 1 |
| *Prevotella copri* | 0 | 4 | 4 |
| *Prevotella oris* | 0 | 1 | 1 |
| *Prevotella stercorea* | 0 | 3 | 3 |
| *Prevotellamassilia timonensis* | 0 | 3 | 3 |
| *Propionispira arcuata* | 0 | 1 | 1 |
| *Pseudoflavonifractor capillosus* | 0 | 2 | 2 |
| *Pseudomonas lini* | 0 | 2 | 2 |
| *Rothia endophytica* | 0 | 1 | 1 |
| *Ruminiclostridium cellobioparum* | 0 | 2 | 2 |
| *Ruminococcus bromii* | 0 | 1 | 1 |
| *Sphaerochaeta coccoides* | 0 | 3 | 3 |
| *Sphingomonas olei* | 0 | 1 | 1 |
| *Streptococcus agalactiae* | 0 | 1 | 1 |
| *Streptococcus porcorum* | 0 | 1 | 1 |
| *Streptococcus suis* | 0 | 1 | 1 |
| *Terrimonas rubra* | 16 | 0 | 16 |
| *Treponema bryantii* | 0 | 1 | 1 |
| *Treponema parvum* | 0 | 2 | 2 |
| *Treponema porcinum* | 0 | 2 | 2 |
| *Ursidibacter arcticus* | 0 | 1 | 1 |
| *Veillonella caviae* | 0 | 1 | 1 |
| *Veillonella parvula* | 0 | 1 | 1 |
